# Detection of dengue group viruses by fluorescence *in situ* hybridization

**DOI:** 10.1186/1756-3305-5-243

**Published:** 2012-10-30

**Authors:** Vincent Raquin, Martin Wannagat, Karima Zouache, Catherine Legras-Lachuer, Claire Valiente Moro, Patrick Mavingui

**Affiliations:** 1 UMR CNRS 5557 Ecologie Microbienne, Université Lyon 1, 43 boulevard du 11 Novembre 1918, Villeurbanne cedex, 69622, France; 2 PRABI, Pôle Rhône-Alpes de Bioinformatique, Villeurbanne, France; 3 Institut Pasteur, Department of Virology, Arboviruses and Insect Vectors, 25–28 rue du Dr Roux, Paris cedex 15, 75724, France

**Keywords:** Dengue, Virus, Fluorescence *In Situ* Hybridization, *Aedes albopictus*

## Abstract

**Background:**

Dengue fever (DF) and dengue hemorrhagic fever (DHF) represent a global challenge in public health. It is estimated that 50 to 100 million infections occur each year causing approximately 20,000 deaths that are usually linked to severe cases like DHF and dengue shock syndrome. The causative agent of DF is dengue virus (genus *Flavivirus*) that comprises four distinct serotypes (DENV-1 to DENV-4). Fluorescence *in situ* hybridization (FISH) has been used successfully to detect pathogenic agents, but has not been implemented in detecting DENV. To improve our understanding of DENV infection and dissemination in host tissues, we designed specific probes to detect DENV in FISH assays.

**Methods:**

Oligonucleotide probes were designed to hybridize with RNA from the broadest range of DENV isolates belonging to the four serotypes, but not to the closest *Flavivirus* genomes. Three probes that fit the criteria defined for FISH experiments were selected, targeting both coding and non-coding regions of the DENV genome. These probes were tested in FISH assays against the dengue vector *Aedes albopictus* (*Diptera*: *Culicidae*). The FISH experiments were led *in vitro* using the C6/36 cell line, and *in vivo* against dissected salivary glands, with epifluorescence and confocal microscopy.

**Results:**

The three 60-nt oligonucleotides probes DENV-Probe A, B and C cover a broad range of DENV isolates from the four serotypes. When the three probes were used together, specific fluorescent signals were observed in C6/36 infected with each DENV serotypes. No signal was detected in either cells infected with close *Flavivirus* members West Nile virus or yellow fever virus. The same protocol was used on salivary glands of *Ae. albopictus* fed with a DENV-2 infectious blood-meal which showed positive signals in the lateral lobes of infected samples, with no significant signal in uninfected mosquitoes.

**Conclusion:**

Based on the FISH technique, we propose a way to design and use oligonucleotide probes to detect arboviruses. Results showed that this method was successfully implemented to specifically detect DENV in a mosquito cell line, as well as in mosquito salivary glands for the DENV-2 serotype. In addition, we emphasize that FISH could be an alternative method to detect arboviruses in host tissues, also offering to circumvent the discontinuity of antibodies used in immunofluorescent assays.

## Background

Dengue fever (DF) is the most common arthropod-borne human viral disease, affecting populations of mainly tropical and sub-tropical countries. The geographic area of DF transmission has expanded considerably during recent decades, and the number of cases reported has increased more than 30-fold since the 1960s [1]. More than 50 million people are infected annually and a third of the world population is at risk of contracting the disease [2].

DF and dengue hemorrhagic fever (DHF) are caused by dengue virus (DENV), an arbovirus (arthropod-borne virus) belonging to the *Flavivirus* genus that comprises other medically important viruses such as yellow fever virus (YFV), West Nile virus (WNV), tick-borne encephalitis virus (TBEV) and Japanese encephalitis virus (JEV) [3]. The DENV nucleocapsid contains a single-stranded, positive-sense RNA genome of approximately 11 kb in length. Four antigenically distinct serotypes (DENV-1 to DENV-4) are known, defined according to cell-surface antigens detected using various serological methods [4,5]. Nucleic acid sequencing showed that within serotypes, genetically distinct groups called genotypes exist whose sequences diverge at given regions of the genome [6].

DENV are maintained in nature within two cycles. In the sylvatic cycle, non-human primates are the reservoirs and the virus is transmitted by arboreal mosquito vectors, such as *Aedes furcifer* and *Aedes luteocephalus* who have been identified as probable vectors in Senegal [7-9]. The human cycle involves highly anthropophilic vectors, mostly *Aedes aegypti* and *Aedes albopictus*, with humans as the only known reservoir and amplification host [10,11]. Tools are available to study DENV, including virus isolation that allows retrieval of particles from host samples [12], and quantitative RT-PCR to identify serotypes or genotypes and estimate virus density [13]. In addition, serological assays were developed to diagnose DENV infection among vertebrate hosts [14]. However, these methods did not allow direct visualization of DENV RNA or particles inside infected samples. Thus, they need to be completed with an *in situ* detection to fully understand the viral infection process and to explore the temporal and spatial tropism of DENV in hosts and reservoirs. Standard DENV *in situ* detection relies on direct targeting of virus particles using serotype-specific antibodies [15]. In humans, immunological detection of DENV in samples obtained from DF/DHF patients revealed virus tropism for liver, spleen, kidney, lung and phagocytic cells [16]. In mosquitoes, an immunodetection approach made it possible to decipher the DENV time course, called the extrinsic incubation period (EIP), time between ingestion of an infectious blood-meal to the infection of the salivary glands and release of the virus into the saliva [17].

Fluorescence *in situ* hybridization (FISH) is a detection method classically employed to target nucleic acids (RNA and DNA) within cells and tissues using a fluorochrome-labelled probe complementary to the target that is then observed by fluorescence microscopy [18]. The increasing range of available fluorochromes, the development of new bioinformatics tools assisting probe design, and improvements in fluorescence microscopy and imaging, have contributed to the extensive application of FISH in monitoring microbes in complex environments [19]. One advantage of FISH, along with accuracy and adaptability, is the possibility to combine several oligonucleotide probes. FISH and derivative techniques are therefore ideal when it is necessary to detect multiple target microorganisms in the same samples [20]. In addition, FISH can be combined to standard detection methods like Indirect Immunofluorescence Antibody Assay (IFA), thus providing a better detection of targets [21,22]. It also represents a useful alternative when antibodies are unavailable or inefficient for a given organism. Few studies have described the use of FISH techniques to monitor different virus taxa [23-25] and no probes are as yet available for DENV. For these reasons, the aim of the study was to develop a FISH-based method, from the design of oligonucleotide probes to the final detection of the four DENV serotypes, including the widest possible range of DENV isolates.

## Methods

### Ethics statement

Anti-DENV hyper-immune ascetic fluid, used as primary antibodies in virus titration assays, was produced in accordance with French and European regulations on care and protection of laboratory animals at the Institut Pasteur that has accreditation from the French Ministry of Agriculture [see permit numbers at http://webcampus.pasteur.fr/jcms/c_97619/agrements-des-animaleries]. This study was approved by the relative IACUC at the Institut Pasteur.

### Probe design

Probe design was based on the method of Jabado *et al*. [26], modified as follows. Briefly, the 3' and 5' untranslated regions (UTR), and coding regions of the DENV genome were recovered from GenBank (release 101, issue date 28, September 2009) [27]. Pfam-A was used to group the selected protein sequences into families according to domain similarity [28]. NCR sequences or those not grouped in Pfam-A families were clustered by CD-Hit [29] at a similarity threshold of 80%. For protein sequence motifs, the most conserved non-overlapping regions of 23 amino acids in length were selected using the statistics of the hidden Markov model and the motifs were mapped to their underlying nucleic sequences. From the resulting database, sequences of candidate probes were shortlisted using the following criteria to maximize specificity: melting temperature ≥ 65°C; GC content between 40% and 60%; no homopolymer of 5 or more of the same nucleotide; no repeats of 10 or more nucleotides; and stem loop length ≤ 11nt. The specificity of all selected sequences was checked using the Basic Local Alignment Search tool (BLAST) querying the NCBI nucleotide database [30]. To further optimize hybridization, only sequences (maximum length, 60 nucleotides) showing a 75% overall similarity or forming 15 consecutive base pairs with a target sequence were synthesized (Life Technologies, Saint-Aubin, France). Entropy, cross-hybridization, and the absence of possible secondary structure, like hairpins, were checked using the online tool DINAMELT [31].

### Viruses

The dengue strains DENV-1 FGA/89, DENV-2 Jam, DENV-3 PaH88/881 and DENV-4 63632 were obtained from the Centre de Ressources Biologiques de l’Institut Pasteur (CRBIP, Paris, France). DENV-2 Bangkok, YFV and WNV strains were provided by the Virology Department of Institut Pasteur (Paris, France). Details of the virus strains are listed in Table 1. Viral stocks were produced on *Aedes* cell lines as previously described [32-36]. 

**Table 1:** **Strains of *Flavivirus *used in this study**

**Genus**	**Virus**	**Strain**	**Locality**	**Year**	**Sample**	**GenBank accession number**	**Reference**
*Flavivirus*	Dengue serotype 1	FGA/89	French Guiana	1989	Human	AF226687.2	[32]
	Dengue serotype 2	Jam/N.1409	Jamaica	1983	Human	M20558.1	[33]
	Dengue serotype 2	Bangkok	Thailand	1974	Human	Unavailable	[34]
	Dengue serotype 3	PaH881/88	Thailand	1988	Human	S67858.1	[35]
	Dengue serotype 4	63 632	Senegal	1983	Human	Unavailable	[36]
	Yellow fever virus	Unavailable	Senegal	1979	Human	Unavailable	[37]
	West Nile virus	Unavailable	France	2001	Horse	AF418554.1	[38]

### Cell culture and virus infection

The C6/36 cell line derived from *Aedes albopictus* larvae [39] was grown at 28°C in Leibovitz’s 15 (L-15) medium (Life Technologies) containing 10% foetal bovine serum (FBS) (PAA, Les Mureaux, France), 50 units/mL penicillin, 50 μg/mL streptomycin and 1X [7.5 mg/L Glycine, 8.9 mg/L L-Alanine, 13.2 mg/L L-Aspargine, 13.3 mg/L L_Aspartic acid, 14.7 mg/L L-Glutamic acid, 11.5 mg/L L-Proline, 10.5 mg/L L-Serine] non essential amino acids (Life Technologies). Twenty-four hours before virus infection, 5 × 10^5^ cells were allowed to attach to a sterile cover slip in shell vials (Sterilin, Newport, United Kingdom). Cell samples were then infected in triplicate with each DENV serotype or YFV and WNV strains at a multiplicity of infection (MOI) of 3. Uninfected cells were used as negative controls. Virus stocks were diluted in appropriate volumes of L-15 medium supplemented with 2% FBS, and layered onto 80%-confluent cell monolayers for 1 hr at 28°C. After virus adsorption the inoculum was removed, 1 mL of medium with 2% FBS was added, and cells were incubated at 28°C. At 5 days post-infection (pi), supernatants were harvested and stored at −80°C for virus titration. Cells were washed once with PBS and fixed for 20 min at room temperature in freshly prepared 4% formaldehyde (Sigma-Aldrich, Lyon, France) in PBS, then rinsed three times in PBS.

To control the replication of the different virus strains used, 1 × 10^6^ cells were inoculated into 6-well plates and incubated at 28°C two days before infection. Virus infection in duplicate at a MOI of 3 was done as described above.

### Mosquito rearing and oral infection

The mosquito *Ae. albopictus* ALPROV originating from La Reunion was reared in standard conditions as reported [40]. For experimental infections, 1 mL of DENV-2 suspension (Bangkok strain) was mixed with 2 mL of washed rabbit erythrocytes (New Zealand White, Charles River) supplemented with ATP at final concentration of 5 mM (Sigma-Aldrich). Seven-day-old female mosquitoes, starved for 24h, were fed with infectious blood at a titer of 10^7.5^ FFU (Fluorescent Focus Units) per mL from a glass feeder as described [41]. Females fed with non-infected blood were used as controls. Fully engorged females were transferred to cardboard containers at 28 ± 1°C and fed with 10% sucrose. At day 14 pi, females were sacrificed and surface-disinfected in 70% ethanol and rinsed twice in sterile PBS. Salivary glands were removed from insects by dissection under a binocular microscope then deposited on a sterile glass cover slip previously coated with 40 μL of 0.01% poly-L-lysine (Sigma-Aldrich) in PBS. Salivary gland samples were fixed for 20 min in freshly prepared 4% formaldehyde in PBS in a shell vial, then washed 3 times in PBS and hybridized to probes as described below.

### Fluorescence *in situ* hybridization

PBS was removed from fixed cells or tissues (as above) and 1 mL of hybridization buffer (5X [750 mM NaCl, 75 mM Na-citrate, pH 7] SSC (Euromedex), 50% formamide, 200 mg/mL dextran sulfate, 250 μg/mL poly(A), 250 μg/mL salmon sperm DNA, 0.1 M dithiothreitol (DTT), 0.5X Denhardt’s solution (Sigma-Aldrich), 250 μg/mL tRNA) containing 10 ng of each DENV probe, labeled at the 5’ end with AlexaFluor® 488, was added. After incubation, the hybridization buffer was discarded and the samples were rinsed once in 1X [150 mM NaCl, 15 mM Na-citrate, pH 7] SSC, 10 mM DTT at room temperature. Further washes were done at 55°C for 15 min each, twice in 1X [150 mM NaCl, 15 mM Na-citrate, pH 7] SSC, 10 mM DTT and twice in 0.5X [75 mM NaCl, 7.5 mM Na-citrate, pH 7] SSC, 10 mM DTT. Finally, samples were rinsed three times in PBS, and each coverslip was carefully taken from the shell vial and mounted on a glass slide with 2.5 μL of DAPI (4’, 6’-diamidino-2-phenylindole, dihydrochloride) at 1 μg/mL in glycerol/PBS (v/v). Slides were stored overnight at 4°C in the dark before microscopic observation.

### Fluorescence microscopy

Slides were viewed under an epifluorescence (Axio Imager Z1, Zeiss) or a confocal microscope (LSM510, Zeiss). Exposure time for each laser (to excite DAPI and AlexaFluor® 488) was standardized among samples. Images for each laser were taken separately then merged using the free-access software ImageJ MacBiophotonics (release 1.46, 32-bit, downloaded from http://www.rsb.info.nih.gov/ij/).

### Virus titration

Fluorescent focus assays (FFA) were used to estimate virus infectious titer on C6/36 cells. Two days before infection, 3 × 10^5^ cells were placed in 96-well microplates and allowed to grow at 28°C. Confluent monolayers were infected with 50 μL of 10-fold serial dilutions of viral suspension in L-15 medium supplemented with 2% FBS. To allow viral adsorption, the microplates were incubated for 1 h at 28°C in 2% FBS L-15 medium with gentle shaking every 15 min. Then an overlay medium (150 μL) composed of L-15 medium with 10% (v/v) FBS and 3.2% (v/v) carboxymethyl cellulose (VWR, Pessac, France), was added. After 5 days at 28°C, cells were fixed by overlaying 100 μL of 4% formaldehyde in PBS for 20 min at room temperature. All the suspension was then gently removed and fixed cells were washed three times in PBS. For primary antibody staining, cells were incubated with mouse hyper-immune ascetic fluid (diluted 1:100 in PBS) specific to each virus for 45 min at 37°C. After three washes with PBS, anti-mouse IgG FITC conjugate (Bio-Rad, Marnes-La-Coquette, France), diluted 1:80 in PBS, was added for 45 min at 37°C. Cells were then rinsed three times in PBS and observed with an inverted fluorescent microscope (Zeiss) equipped with a FITC filter. Fluorescent foci of infected cells were counted under a 10× objective and virus titers were expressed as the number of FFU per mL [42].

### RNA extraction, reverse transcription and diagnostic PCR

At 5 days pi, supernatants from infected cells in 6-well plates were removed. Cells were harvested and pelleted using a tabletop centrifuge, then stored at −80°C until use. Total RNA was obtained using the Qiagen RNeasy Mini Kit (Qiagen, Courtaboeuf, France), according to the manufacturer’s recommendations, first crushing the cell pellet with a pestle for 30 s in 350 μL RLT buffer. Residual DNA was eliminated using the Turbo DNA-free kit as recommended by the manufacturer (Life Technologies).

RNA (100 ng) was reverse transcribed using 400 U SuperScript III reverse transcriptase (Life Technologies) and 200 ng of random primers in a 50-μL reaction volume according to the manufacturer’s recommendations. The resulting cDNA samples were treated with 10 U/μL RNase H (Life Technologies) for 20 min at 37°C, then purified using a QIAquick PCR purification kit according to manufacturer’s instructions (Qiagen).

To detect virus RNA, diagnostic PCR was done with *Flavivirus*-specific primers PF1S (5’-TGYRTBTAYAACATGATGGG-3’) and PF2R-bis (5’-GTGTCCCAICCNGCNGTRTC3’) targeting an approximately 230-bp fragment of the NS5 gene [43]. The 25-μL reaction mixture contained 40 ng of cDNA template, 0.5 U *Taq* polymerase, [20 mM Tris–HCl (pH 8.4), 500 mM KCl] reaction buffer (both from Life Technologies), 1.5 mM MgCl_2_, 200 μM of each dNTP and 200 nM of each primer. PCR was performed in a T1 Thermocycler (Biometra) as follows: 95°C for 3 min; 40 cycles of 94°C for 1 min, 50°C for 1 min, 72°C for 1 min; and 72°C for 10 min. PCR products were checked by electrophoresis through 1% agarose gel alongside a low-range DNA ladder (Fermentas, Courtaboeuf, France). Fragments of expected sizes were sequenced (Sanger sequencing).

## Results and discussion

### *In silico* analysis of selected DENV probes

The probe design strategy allowed a set of ten probes that fitted the criteria listed above to be obtained. We selected two probes with a GC content close to 50% and a lower number of hairpins, while targeting the broadest range of DENV isolates. Two oligonucleotide probes, designated DENV-ProbeA and DENV-ProbeB, which target respectively a fragment of a 3’ untranslated region (UTR) and a fragment of the non-structural 3 (NS3) protein-encoding gene were first selected (Table 2). When analyzed against the NCBI nucleotide collection using BLASTn, with a word size of 7, an expected threshold of 1,000 and a minimal e-value of 0.05, the DENV-Probes A and B sequences matched with all DENV serotypes (Additional file 1: Figure S1). These results indicated that most of the sequences for each serotype present in GenBank would be detected by at least one of the two probes, except for DENV-4. Indeed, the specificity of the two probes for the DENV-4 serotype was relatively low, and matched few DENV-4 isolates. Although this result might be unexpected as there is relatively little genomic divergence between DENV serotypes [44], we found that DENV-4 is the serotype with the fewest sequences available in GenBank, which could partly explain the BLASTn results. To detect as many DENV isolates as possible, the DENV-ProbeC, which targets a fragment of the non-structural 2 (NS2) protein-encoding gene and matches with all sequenced complete genomes of DENV-4 (Table 3, Additional file 2: Figure S1), was added to the initial set of probes. 

**Table 2:** DENV probe sequences

	**Sequence (5’ to 3’)**	**GC%**	**Tm (°C)**	**Hairpin DG**
DENV-ProbeA	GCCGGATTAAGCCATAGTACGGTAAGAGCTATGCTGCCTGTGAGCCCCGTCTGAGGACGT	57	56.7	−2
DENV-ProbeB	AGAAAATGACCAGTACATATTCACGGGCCAGCCTCTCAACAATGACGAGGACCATGCTCA	48	48.3	−1.8
DENV-ProbeC	ATGGTGCTTCACTGGGGAAAGAAATAACCAAATTCTAGAAGAAAACATGGAGGTTGAAAT	37	36.7	−0.9

**Table 3:** Number of isolates targeted by the three DENV probes simultaneously

	**DENV-1**	**DENV-2**	**DENV-3**	**DENV-4**
Isolates^a^ (%)	1279 (98.8)^b^	885 (99.7)^b^	654(99.8)^b^	97 (100)^b^
e-value max	9x10^-23^	9x10^-5^	9x10^-23^	9x10^-23^
e-value min	1x10^-3^	3x10^-3^	1x10^-14^	1x10^-21^
Query max	100	98	100	100
Query min	90	85	98	98
Identity max	100	100	100	100
Identity min	98	94	98	73

^a^Complete genome sequences only.

^b^NCBI GenBank nucleotide database, release 189 of 15^th^ April 2012.

Using the BLASTn parameters described above, the three DENV probes were matched against the YFV (taxid: 11089), WNV (taxid: 11082), JEV (taxid: 11072), chikungunya virus (CHIKV, taxid: 337124) and *Aedes* (taxid: 7158) and no sequences were retrieved. These results indicated that *in silico*, no hybridization with these viruses could occur.

### Detection of DENV serotypes in infected cell cultures

**Figure 1:**
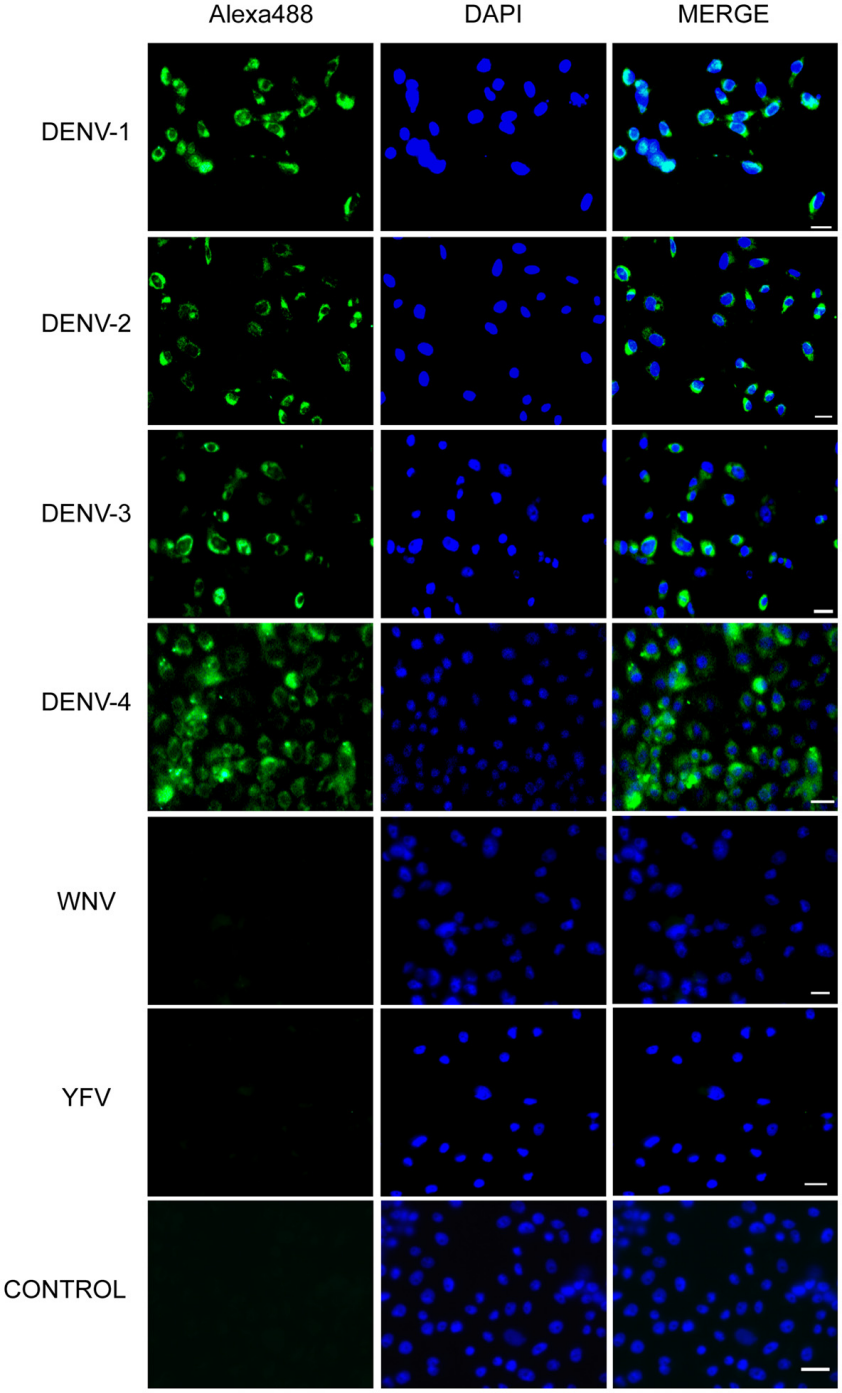
**Specific detection of DENV without cross-hybridization towards closely related *Flavivirus.*** Cells were infected with the most closely related *Flavivirus* YFV and WNV. DENV-1, DENV-2, DENV-3, DENV-4 were used as positive controls. FISH was performed using the three selected probes simultaneously at day 5 post-infection. Positive hybridizing signals (green) are seen in the cytoplasm of C6/36 cells infected with DENV-1 to DENV-4. No hybridizing signals were visible in cells infected with YFV and WNV or in uninfected cells. Host cell nuclei are stained with DAPI. Bar, 10 μm.

The FISH method using the three DENV-ProbeA, DENV-ProbeB, and DENV-ProbeC probes together was first tested using the well-characterized arbovirus-permissive *Ae. albopictus* C6/36 cell line. The use of three probes simultaneously may lead to a considerable increase in the hybridizing signal, with no incidence on the level of the background signal [45]. After infection at a MOI of 3, green fluorescent dots corresponding to the hybridized probes with DENV RNA were observed in the cytoplasm of cells infected with each of the four DENV serotypes at 5 days pi (Figure 1). No green fluorescent signal was observed in the cytoplasm of uninfected control cells. Nuclei were strongly stained blue with DAPI with, in some cases, a faint green autofluorescence (Figure 1). This hybridization pattern was expected as members of the genus *Flavivirus* are known to replicate almost exclusively in the cytoplasm of infected cells [46,47]. Overall, these observations validate the choice of probes and the hybridization protocol. 

To assess probe specificity towards DENV, we also infected cells with genetically closely related *Flavivirus* YFV and WNV, then hybridized with the three DENV-ProbeA, DENV-ProbeB, and DENV-ProbeC probes together as described above. As expected from *in silico* analyses, no green fluorescence was detected at day 5 pi, as was observed for uninfected cells (Figure 1). *Flavivirus* replication kinetics in C6/36 is known to reach the plateau phase at 5 or 6 days pi [48,49]. This shows that the DENV probes do not readily cross-hybridize with the most closely related viruses in insect cells.

**Figure 2:**
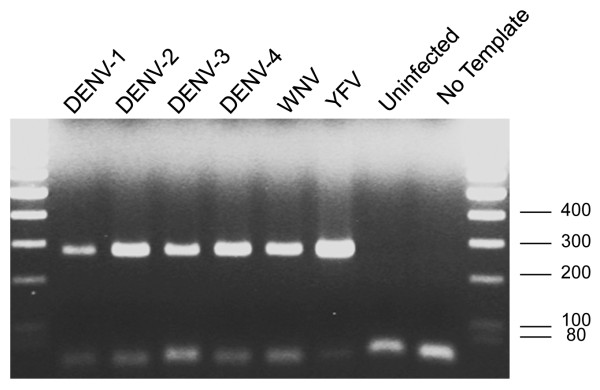
**Diagnostic PCR of viral RNA in C6/36 cells.** C6/36 cells were infected with DENV-1 to DENV-4, YFV or WNV and 5 days later RNA was extracted from cells and reverse transcribed. PCR was performed on the cDNA obtained to amplify a 230-bp fragment of the NS5 gene, conserved among *Flavivirus*. Fragments of the expected size were obtained for all the six viruses, but not for uninfected cells. Ladder Mass Ruler DNA Low Range (Fermentas) was used as size marker (bp).

To ensure that the absence of signal in the above YFV and WNV samples was not an artifact of defective viral infection, supernatants from the same cell cultures previously used in FISH were titrated for viruses. The infectious titers reached 5.6 × 10^11^ FFU/mL for WNV, 2.2 × 10^11^ FFU/mL for YFV and those of DENV1 to DENV-4 were comprised between 1.4 to 2.0 x 10^5^ FFU/mL (positive controls). No fluorescent foci were counted for uninfected cell supernatants. In addition, the presence of viral RNA was ascertained by reverse transcription and PCR amplification using genus-specific primers. Bands of the expected size (230 bp) were obtained for all the six viruses from day 5 pi cultures (Figure 2) and sequencing of PCR products (data not shown) confirmed they belonged to the respective viruses tested, indicating that replication of each virus was productive in C6/36 infected cells.

### Monitoring of DENV in mosquito tissues

The virus infection process in mosquitoes is very different from that in cell culture. To evaluate the usefulness of the DENV FISH probes, they were used to localize DENV in host tissues. Females of *Ae. albopictus* ALPROV from La Reunion fed with a DENV-2 Bangkok strain infectious blood-meal were dissected at 14 days pi. The DENV probes were designed to match with the largest number of DENV isolates as possible, within each serotype. As a consequence, we decided to use the Bangkok isolate of DENV-2 (Table 1) to infect *Ae. albopictus*, as we had experimental data on it’s replication kinetic in this mosquito strain system in contrast to previously used DENV strains [42,50].

**Figure 3:**
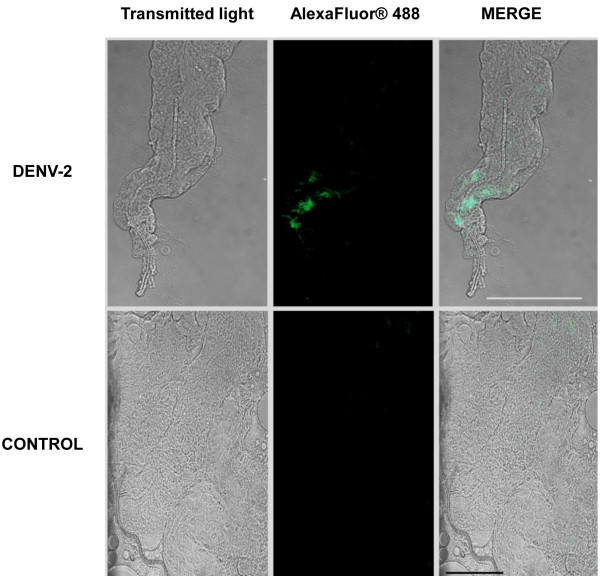
**FISH detection of viral RNA in salivary glands of *Aedes albopictus *by confocal microscopy.** FISH was carried out on salivary glands dissected from *Ae. albopictus* females at 14 days post-infection with blood-meals containing DENV-2 Bangkok strain, using the three DENV-Probes simultaneously. Viral RNA is detected (green) in the distal zone of the lobe close to the salivary duct. No signal was detected in control females engorged with an uninfected blood-meal. Bar, 500 μm. A global view of salivary glands is provided in the supplementary Figure S2.

The dissected salivary glands of infected females were hybridized with the three DENV-Probes, as described. Confocal microscopic images showed dots indicative of probe hybridization in the lateral lobes of DENV-infected females at 14 days pi (Figure 3). In contrast, no significant fluorescent signals were observed in organs of uninfected mosquitoes used as negative controls (Figure 3). This result confirmed that our probes could be used to detect DENV-2 in *Ae. albopictus* salivary glands after oral infection of the vector.

In the principal DENV vector *Ae. aegypti*, a period of 7 to 10 days post-infection is necessary to reach maximum viral replication, allowing the virus particles to invade salivary glands [17]. *Ae. albopictus* is less competent for DENV, the incubation period to reach high RNA levels is longer and close to 14 days [50,51]. Moreover, the density of infectious DENV particles in the salivary glands was found to be weak in *Ae. albopictus* ALPROV strain, due to *Wolbachia* infection (Mousson *et al.,* submitted). Despite this constraint, we were able to detect viral RNA in *Ae. albopictus* salivary glands at 14 days pi. This result shows that FISH is an efficient method that can be used even with complex material like mosquito organs.

To detect DENV *in situ*, the indirect Immunofluorescence Antibody Assay (IFA) is the standard method, with serotype specific antibodies commercially available [52]. However, IFA method targets antigenic proteins at the surface of viral particles whereas the FISH technique is based on the detection of viral RNA, two different labels of the viral cycle. The FISH method allows monitoring of DENV replicating genome during infection process and may represent a complementary method to IFA. Moreover, the literature provides numerous studies using FISH and IFA in combination [22,53,54]. In addition, we emphasize that FISH could be an alternative method to detect arboviruses in host tissues, also offering to circumvent the discontinuity of antibodies used in immunofluorescent assays.

## Conclusions

In this study, oligonucleotide FISH was developed and used for the first time to detect replicating genomic DENV RNA. Viral RNAs were detected in both infected cell lines and salivary glands of the mosquito vector *Ae. albopictus*. As mentioned, *Ae. albopictus* is a less efficient vector of DENV than *Aedes aegypti*. Recent work has established that *Wolbachia*, a bacterial endosymbiont naturally present in *Ae. albopictus*, influences vector competence by interfering with viral infection and dissemination in mosquitoes [40,55]. This FISH approach could be useful to identify DENV tropism in the less efficient vector *Ae. albopictus*. Furthermore, it could help to explain the bacterial influence on DENV dissemination, especially as we have already detected *Wolbachia* in mosquito tissues using the same FISH-based protocol presented here [56]. This could improve our comprehension of mosquito vectorial competence for DENV.

DENV replication begins with the synthesis of a negative intermediate RNA, which appears transiently double-stranded RNA (dsRNA ) for production of positive strands used in virion assembly [57]. dsRNA is considered as the true replicative intermediate form of DENV, and can be detected with specific antibodies [58]. The FISH technique could be used to discriminate positive and negative strands by designing specific probes, and eventually couple to IFA in order to detect every intermediate as well as viral proteins [59].

Cases of co-infection with multiple DENV serotypes [60] and chikungunya virus were reported in human [61,62] and in *Ae. albopictus*[63], the latter having the ability to simultaneously deliver DENV and CHIKV in its saliva [63]. These multiple infections, together with potential multipartite interactions, emphasize the importance in some circumstances of monitoring more than one infectious agent at a time [64]. This work provides a method to design oligonucleotide probes specific for a given DNA/RNA template, which may be useful for microorganisms for which no antibodies are available yet, as well as a unique hybridization protocol that has already been used to detect other types of microorganisms [56]. In this context, FISH could be an adapted alternative method for monitoring multiple biological agents in a given tissue.

## Abbreviations

Arbovirus: Arthropod borne virus; DAPI: 4’, 6’-diamidino-2-phenylindole, dihydrochloride; DENV: Dengue virus; dsRNA: Double-stranded RNA; DTT: Dithiothreitol; EIP: Extrinsic incubation period; FBS: Foetal bovine serum; FFA: Fluorescent focus assay; FFU: Fluorescent forming unit; FISH: Fluorescence *In Situ* Hybridization; IFA: Indirect immunofluorescence antibody assay; JEV: Japanese encephalitis virus; L-15: Leibovitz’s 15 medium; LTR: Long terminal repeat; MOI: Multiplicity of infection; NCR: Non coding region; PBS: Phosphate buffered saline; PI: Post infection; SSC: Saline-sodium citrate; TBEV: Tick-borne encephalitis virus; UTR: Untranslated region; WNV: West-nile virus; YFV: Yellow fever virus.

## Competing interests

The authors declare that they have no competing interests.

## Author’s contributions

VR, CVM, and PM conceived the work. VR, MV, KZ, CVM and PM designed and performed the experiments. VR, MV, KZ, CLL, CVM and PM analyzed the results. VR, CVM, PM wrote the paper in collaboration with other authors. All authors read and approved the final version of the manuscript.

## Supplementary Material

Additional file 1: Figure S1 Alignment of DENV-Probes on genomic RNA sequence of DENV isolates. ClustalW Multiple Alignment was performed to align DENV-ProbeA, DENV-ProbeB and DENV-ProbeC against some well-targeted DENV RNA sequences. As mentioned in the text, very few isolates of DENV-4 were targeted by probes A and B. The mismatch numbers for DENV-ProbeA with DENV-4 were high and distorted alignment, so DENV-4 was removed from the analysis. As the DENV-ProbeC specifically targets serotype 4, only alignment on DENV-4 sequence isolates is presented. Click here for file

Additional file 2: Figure S2 Epifluorescence microscopy showing morphology of *Aedes albopictus* salivary glands. The cell nuclei appear in green after labelling by SYTOX. SD, salivary duct; LL: Lateral Lobe; ML: Median Lobe. Bar, 500 μm. Click here for file
