# Supplementary figures and images for: Detection of dengue group viruses by fluorescence in situ hybridization
Source: Parasit Vectors. 2012 Oct 30;5:243. doi: 10.1186/1756-3305-5-243 (PMC3507901; doi:10.1186/1756-3305-5-243)

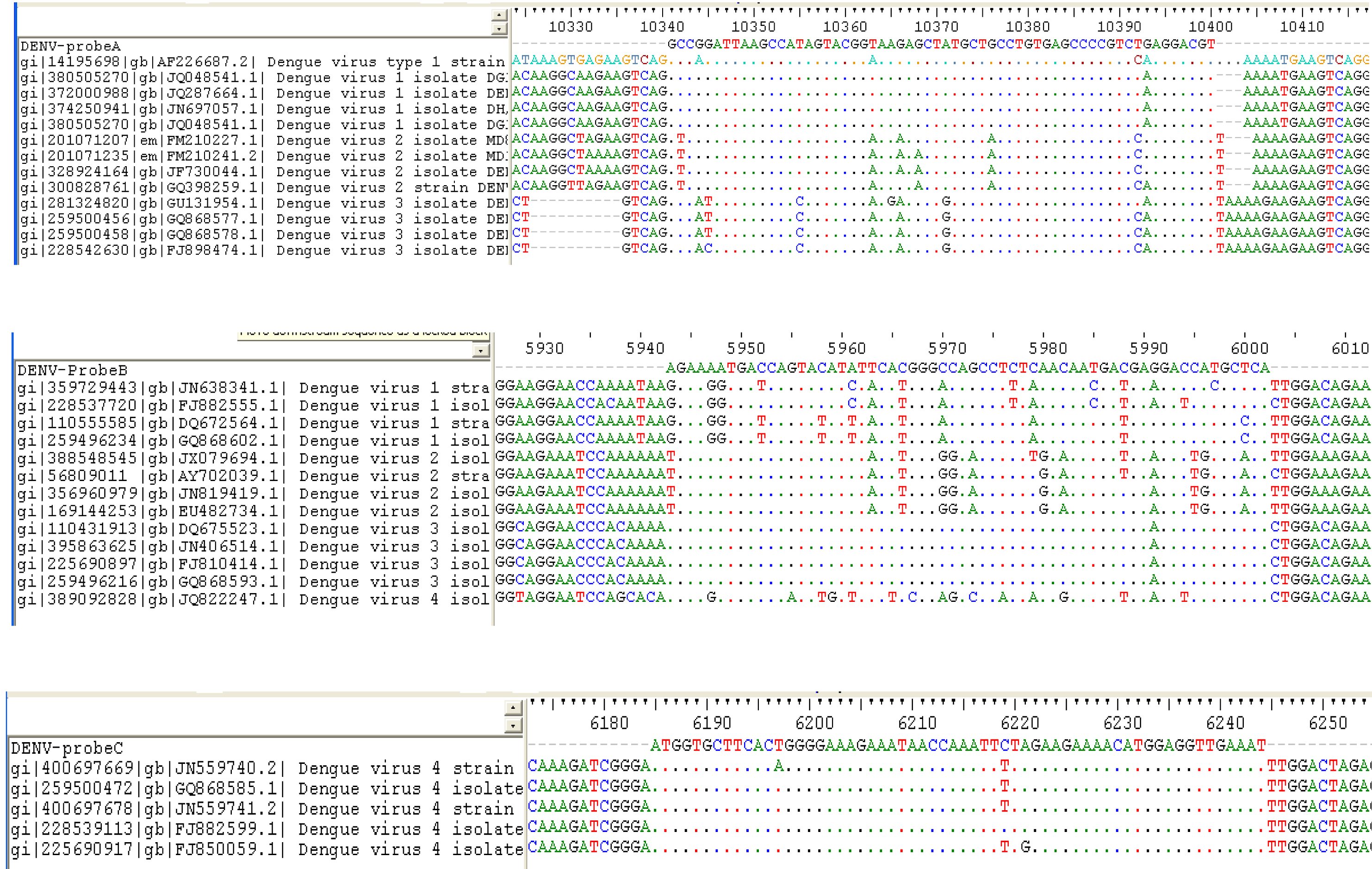

Supplement: Additional file 1: Figure S1 — Alignment of DENV-Probes on genomic RNA sequence of DENV isolates. ClustalW Multiple Alignment was performed to align DENV-ProbeA, DENV-ProbeB and DENV-ProbeC against some well-targeted DENV RNA sequences. As mentioned in the text, very few isolates of DENV-4 were targeted by probes A and B. The mismatch numbers for DENV-ProbeA with DENV-4 were high and distorted alignment, so DENV-4 was removed from the analysis. As the DENV-ProbeC specifically targets serotype 4, only alignment on DENV-4 sequence isolates is presented. [file 1756-3305-5-243-S1.tiff]

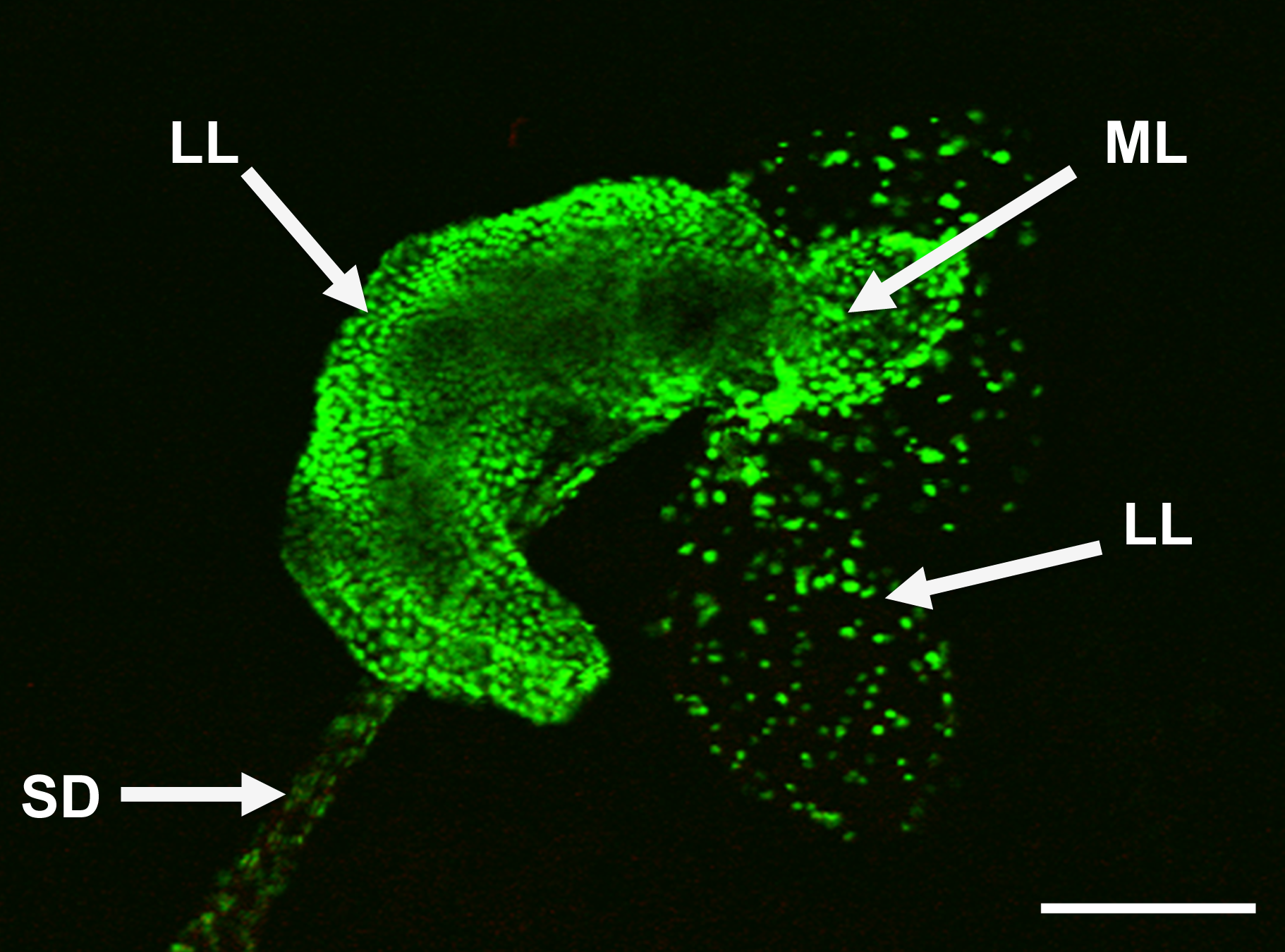

Supplement: Additional file 2: Figure S2 — Epifluorescence microscopy showing morphology of Aedes albopictus salivary glands. The cell nuclei appear in green after labelling by SYTOX. SD, salivary duct; LL: Lateral Lobe; ML: Median Lobe. Bar, 500 μm. [file 1756-3305-5-243-S2.tiff]
